# Topical Cream-Based Dosage Forms of the Macrocyclic Drug Delivery Vehicle Cucurbit[6]uril

**DOI:** 10.1371/journal.pone.0085361

**Published:** 2014-01-15

**Authors:** Marian Seif, Michael L. Impelido, Michael G. Apps, Nial J. Wheate

**Affiliations:** Faculty of Pharmacy, The University of Sydney, Sydney, New South Whales, Australia; University of Maryland, United States of America

## Abstract

The macrocycle family of molecules called cucurbit[*n*]urils are potential drug delivery vehicles as they are able to form host-guest complexes with many different classes of drugs. This study aimed to examine the utility of Cucurbit[6]uril (CB[6]) in topical cream-based formulations for either localised treatment or for transdermal delivery. Cucurbit[6]uril was formulated into both buffered cream aqueous- and oily cream-based dosage forms. The solid state interaction of CB[6] with other excipients was studied by differential scanning calorimetry and the macrocycle's transdermal permeability was determined using rat skin. Significant solid state interactions were observed between CB[6] and the other dosage form excipients. At concentrations up to 32% w/w the buffered aqueous cream maintained its normal consistency and could be effectively applied to skin, but the oily cream was too stiff and is not suitable as a dosage form. Cucurbit[6]uril does not permeate through skin; as such, the results imply that cucurbituril-based topical creams may potentially only have applications for localised skin treatment and not for transdermal drug delivery.

## Introduction

**Figure 1:**
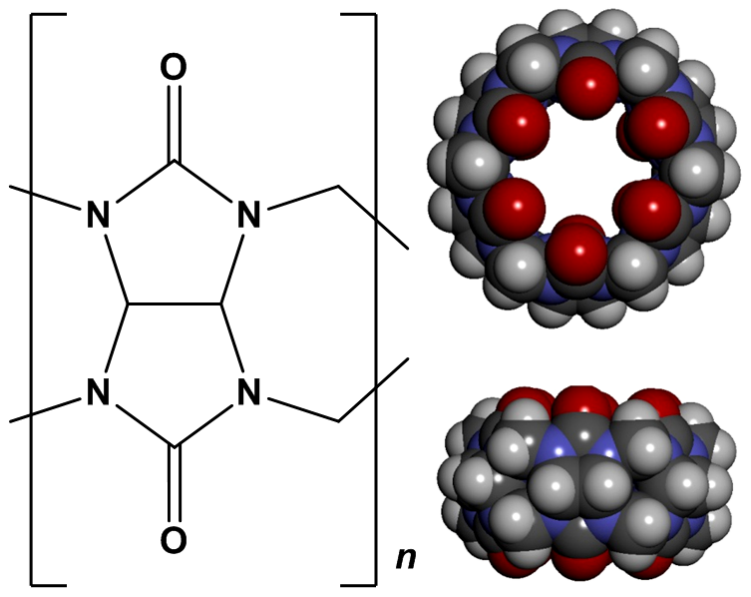
The chemical structure of cucurbit[*n*]urils (*n* = 5, 6, 7, 8, 10 or 14 subunits) and a molecular model of the Cucurbit[6]uril homologue based on its single crystal X-ray structure.

A new family of macrocycles called cucurbit[*n*]urils (Fig. 1, *n* = 5–8, 10, 14) [1]–[5], have demonstrated potential as drug delivery vehicles as they are able to form host-guest complexes with many classes of drug [6]–[13]. The drugs are either partially or wholly encapsulated within the macrocycle's hydrophobic cavity with encapsulation further stabilised through hydrogen bonding and ion-dipole interactions between the drugs and the cucurbituril portals [1], [3], [4], [6].

Their formation into host-guest drug complexes has demonstrated several important applications, including: increased drug solubility [14]–[16], induced drug pKa shifts [17], biorecognition [18]–[20], improved drug stability and reduced drug degradation during manufacture, storage and within the human body [8], [21]–[24], taste masking of orally delivered drugs [8], modification of drugs' pharmacokinetics [25], and finally, investigation of photo-controlled drug release [26]. As drug delivery vehicles, the most commonly used sizes are CB[6], CB[7] and CB[8], as CB[5] is too small to incorporate drugs and CB[10] is too large to adequately bind drugs in its cavity [27]–[30]. As a new cucurbituril homologue, no drug delivery studies of CB[14] have yet been undertaken [5].

To realise the drug delivery potential of cucurbiturils they must be formulated into appropriate dosage forms for human use, but to date there has been little investigation into cucurbituril formulation. Previously we have examined solid oral dosage forms of cucurbituril [7]. Using microcrystalline CB[6], tablets containing up to 50% w/w cucurbituril could be manufactured with suitable tablet hardness, surface smoothness and tablet disintegration times in both simulated gastric and intestinal fluid [7].

Cucurbiturils are known to form host-guest complexes with a range of drugs that have applications in dermal and transdermal delivery. For example, cucurbiturils are able to bind nicotine which has application as a slow release formulation [31]. They are also able to bind local anaesthetics such as procainamide, tetracaine, procaine and prilocaine [32], and can also bind fungicides such as carboxin [33], fuberidazole [34] and thiabendazole [16]. Therefore, as part of a wider cucurbituril dosage formulation project in our group it was of interest to develop topical cream-based formulations of cucurbituril for dermal and transdermal drug delivery.

In this work the utility of CB[6] as an effective excipient in topical formulations was studied using two common base creams from the Australian Pharmaceutical Formulary and Handbook 22^nd^ edition (APF22): buffered cream aqueous (BCA) and oily cream [35]. Dosage forms of both creams were manufactured containing CB[6] and the macrocycle's solid state interactions with the other dosage form excipients was examined. The permeability of CB[6] was examined using rat skin and monitored by fluorescence spectroscopy.

## Materials and Methods

### Materials

Glycoluril, hydrochloric acid (37%), 1,6-dibromohexane, deuterium oxide (99.9%), deuterium chloride (35%), isoquinoline and chlorocresol (4-chloro-3-methyphenol) were purchased from Sigma-Aldrich. Paraformaldehyde was purchased from AlfaAesar. Phenoxyethanol, zinc oxide BP, lanolin anhydrous and citric acid monohydrate were purchased from the Sydney Essential Oil Company. Cetostearyl alcohol was purchased from Bronson and Jacobs. White soft paraffin was supplied by The University of Sydney Dispensing Laboratory. Sodium phosphate BP was purchased from the Government Store Department. 1,4-dioxane, calcium hydroxide solution and sulfuric acid were purchased from Merck. Arachis oil was purchased from Proteco and oleic acid from Professional Compounding Chemists of Australia. All water was obtained from an SG ultra clear water system. Phosphate buffered saline (PBS) solutions were made using pure water and PBS tablets from Sigma Life Sciences. All other chemicals including: methanol, ethanol and diethyl ether, were of analytical reagent grade.

### CB[6] Synthesis and Processing

Cucurbit[6]uril was synthesised and purified using an adaptation of the method of Walker et al. [7]. Glycouril (20 g) was dissolved in sulfuric acid (98%, 110 mL) at 70°C. Paraformaldehyde (8.8 g) was then added and the solution stirred at 70°C overnight. The solution was allowed to cool to room temperature before CB[6] was precipitated by the addition of ∼1700 mL methanol. The solution was left to settle for 1 day before the methanol/acid supernatant was decanted. This purification process was repeated a second time. The solution was then heated to ∼50°C for several hours to aid evaporation of any remaining methanol. Next, the precipitate was dissolved in a minimum amount (∼100–200 mL) of hot concentrated HCl. The solution was then cooled to −20°C to crystallise pure CB[6]. The solution was decanted and the solution placed back in the freezer to allow for further crystallisation. The remaining crystals were collected via vacuum filtration using grade 1 filter paper, washed with water, air-dried for ∼ 1 h and oven-dried at ∼ 100°C for several hours.

### Synthesis of fluorescence guest for tracking CB[6]

The fluorescence marker, 2,2′-(hexane-1,6diyl)diisoquinolinium, was synthesised using an adaptation of the method Fan et al [36]; however, production of the fluorescence marker following this method does not result in a pure product. After collection of the product by filtration at the end of the synthesis and purification method in the paper, the fluorescence marker was then additionally redissolved in a minimum volume of ethanol and left to recrystallise through the slow addition of gaseous diethyl ether. The resultant crystals were collected by vacuum filtration on nylon filter paper (0.2 µm), washed with diethyl ether and air-dried for several hours.

### Fluorescence calibration graph

A series of dilutions from 1 to 10 µM of the free fluorescent marker and the fluorescent marker in CB[6] in water were prepared to obtain calibration graphs. Fluorescence spectra were measured on a Shimadzu RF-5301 PC spectrofluorometer with excitation and emission wavelengths of 338 nm and 377 nm respectively, through a quartz cuvette with a path length of 1 cm.

### Emulsifying ointment preparation

Emulsifying ointment was prepared as described in the APF22 [35]. Emulsifying wax (300 g), white soft paraffin (500 g) and liquid paraffin (200 g) were melted together in a large plastic beaker in a microwave in 1 min bursts. The ingredients were stirred with a spatula until mixed and then cooled to room temperature.

### Preparation of cream formulations - Buffered cream aqueous

Buffered cream aqueous, as described in the APF22, was prepared by mixing a lipophilic and an aqueous phase together at ∼70°C. The aqueous phase was made by dissolving sodium phosphate (2.5 g), citric acid monohydrate (0.5 g) and chlorocresol (0.1 g) in water (∼60 mL) in a glass beaker on a hotplate (>80°C) with stirring. The beaker was covered with a watch glass to reduce solvent evaporation. The lipophilic phase consisted solely of emulsifying ointment (30 g), which was melted using a microwave oven and in a plastic beaker with 10 s bursts of energy. The aqueous phase was then added to the melted emulsifying ointment and immediately mixed with a spatula until it cooled and formed a semi-solid. As the cream formed, the weight was adjusted with water to a final total mass of 100 g. In later experiments, as an alternate preservative, chlorocresol was replaced with phenoxyethanol (1 mL), which was added after mixing both phases.

### Preparation of cream formulations - Oily cream

Zinc cream oily and oily cream samples were produced according to the APF22. Preparation of the zinc cream involved mixing a lipophilic and an aqueous phase together in a mortar and pestle. The mortar was first heated with hot water and then dried thoroughly. Zinc oxide (6.4 g) was added to lanolin anhydrous (3.5 g) in the mortar. The zinc oxide was incorporated carefully into the lanolin anhydrous to ensure even distribution. Arachis oil (4.3 mL) was added slowly using a pipette to dissolve the lanolin anhydrous. Oleic acid (0.1 mL) was then added using a pipette. The lipophilic phase was mixed in the mortar and pestle until all the lanolin dissolved. The aqueous phase, consisting of a calcium hydroxide solution (1% w/v, 6.1 mL), was added to the mortar in several portions and mixed thoroughly after each addition until the solution was completely incorporated. For preparations of cucurbituril-based oily cream formulations, the zinc oxide in the formulation was replaced with CB[6].

### Incorporation and formulation of CB[6] into the creams

Cucurbit[6]uril was formulated into the creams by two methods: either by dissolution in the aqueous phase or incorporation as a fine powder. To aid dissolution, the CB[6] crystals were first ground to a fine powder in a mortar and pestle. To determine the amount of CB[6] that could be dissolved in the aqueous phase of the BCA formulation, CB[6] (added in 50 mg increments until solution saturation) was added to a solution containing sodium phosphate (2.5 g) and citric acid monohydrate (0.5 g) in water (∼60 mL) at >80°C with stirring. Once dissolved, the solution was allowed to cool to room temperature. The solubility was determined by the maximum amount of CB[6] that dissolved in the solution without recrystallisation once the solution had cooled to room temperature.

An alternate method of formulating CB[6] into the creams involved incorporating CB[6] as a fine powder. Cucurbit[6]uril was incorporated into the BCA by the method of doubling to the final cream. In the oily cream, ground CB[6] powder was incorporated into the lanolin anhydrous in the mortar in the initial stages of the cream preparation instead of the excipient normally used, zinc oxide. The arachis oil and oleic acid were then added to the mortar. The lipophilic phase was mixed in the mortar and pestle until all the lanolin anhydrous dissolved. The aqueous phase, which consisted of calcium hydroxide solution, was then added to the mortar in several portions and mixed thoroughly after each addition until the solution was completely incorporated.

### Solid state interactions

Interactions between CB[6] and the other dosage form excipients that are solids at room temperature were studied using differential scanning calorimetry (DSC). A standard Cell 2920 modulated DSC supplied with a liquid nitrogen system at a flow rate of 60 mL/min was used. Individual samples were weighed accurately in Tzero aluminium crucibles with a similar empty crucible used as a reference. Each sample was heated at a rate of 5°C/min from 0 to ∼10°C above the excipient's known melting point. The data were collected and analysed using a Universal V4.2E TA instrument.

### Thermogravimetric Analysis

Thermogravimetric analysis (TGA) was conducted using a Hi-Res TGA 2950 instrument. Citric acid monohydrate and CB[6] samples were weighed on tared platinum pans and heated at a rate of 2°C/min to 120°C and 350°C, respectively. The data were collected and analysed using a Universal V4.2E TA instrument.

### Cucurbit[6]uril skin permeability

Male and female Australian albino white (AAW) rats were obtained previously euthanised with pCO_2_ from the Laboratory Animal Services at The University of Sydney after their use in behavioural projects had been finalised (Animal Ethics Committee approval number L29/5-12/3/5765). The abdominal fur was clipped and carefully shaved (to better represent human skin) using a razor and scalpel before the abdominal skin was then surgically excised. The skin samples were stored in phosphate buffered saline (PBS) solution at 4°C until use. The abdominal area of the excised skin on each rat was measured with a ruler and the average area was calculated. Prior to undertaking skin permeability tests, each piece of skin was restretched to the average length and width it would have been on the animal using a cork board and pins. Any remaining fur on the skin was removed using a scalpel. Samples of each cream containing either free fluorescent marker or CB[6] encapsulated fluorescent marker (40 mg of cream in total per sample) was applied to skin samples on a PermaGear 25 mm Franz cells with a receiver volume of 20 mL. The reservoir was filled with PBS and kept at 37°C by stirring (500 rpm) and immersion in a water bath. Solution samples were taken from the reservoir 1 h after application of the creams to the skin.

## Results and Discussion

The purpose of this work was to assess the utility of cucurbiturils as an effective excipient in topical cream dosage forms for localised and transdermal drug delivery. Cucurbit[6]uril was selected as a representative macrocycle from the cucurbituril family as it is the cheapest and easiest to use for research purposes [7].

### Cucurbit[6]uril processing

Our previous method of processing CB[6] involved several repetitive steps which are time consuming and result in a low yield of CB[6] crystals [7]. This old method involves collecting the CB[6] precipitate by vacuum filtration and then washing it with copious amounts of water. It would then need to be collected and dried under vacuum, and redissolved in a minimum volume of concentrated HCl. The solution would then be left for a period of days to weeks to allow for crystallisation of pure CB[6] crystals. Finally, using this old method, the CB[6] crystals are washed with a HCl/water solution, rinsed with pure water, then collected via vacuum filtration and oven dried at 110°C for several hours.

A new and more efficient method of processing CB[6] was therefore developed to obtain a faster and higher yield of pure CB[6] crystals. This method involved precipitating CB[6] from the acid solution directly after synthesis by the addition of copious amounts of methanol. The solution is left to form a precipitate before the acid/methanol supernatant is decanted. This process is repeated a second time before the precipitate and any small amount of remaining solution are then heated to aid evaporation of any remaining methanol until a sludge-like residue forms. The precipitate is then dissolved in a minimum amount of hot concentrated HCl, the solution cooled to −20°C overnight and the crystals collected via vacuum filtration. This purification method results in a higher yield of pure CB[6] crystals and is also less time consuming since it consists of fewer preparation steps compared with the old method.

### Cucurbit[6]uril-excipient solid state interactions

As a novel excipient in human topical dosage forms, CB[6] may interact positively or negatively with the other excipients in the creams, including chlorocresol, cetostearyl alcohol, white soft paraffin, etc. Differential scanning calorimetry (DSC) can be employed to determine whether CB[6] physically interacts with any of these excipients [7], [37]. Each excipient was first analysed by DSC to attain baseline values. Then 50:50 w/w mixtures of CB[6] with each excipient were prepared by grinding them together in a mortar and pestle and analysed by DSC.

**Figure 2:**
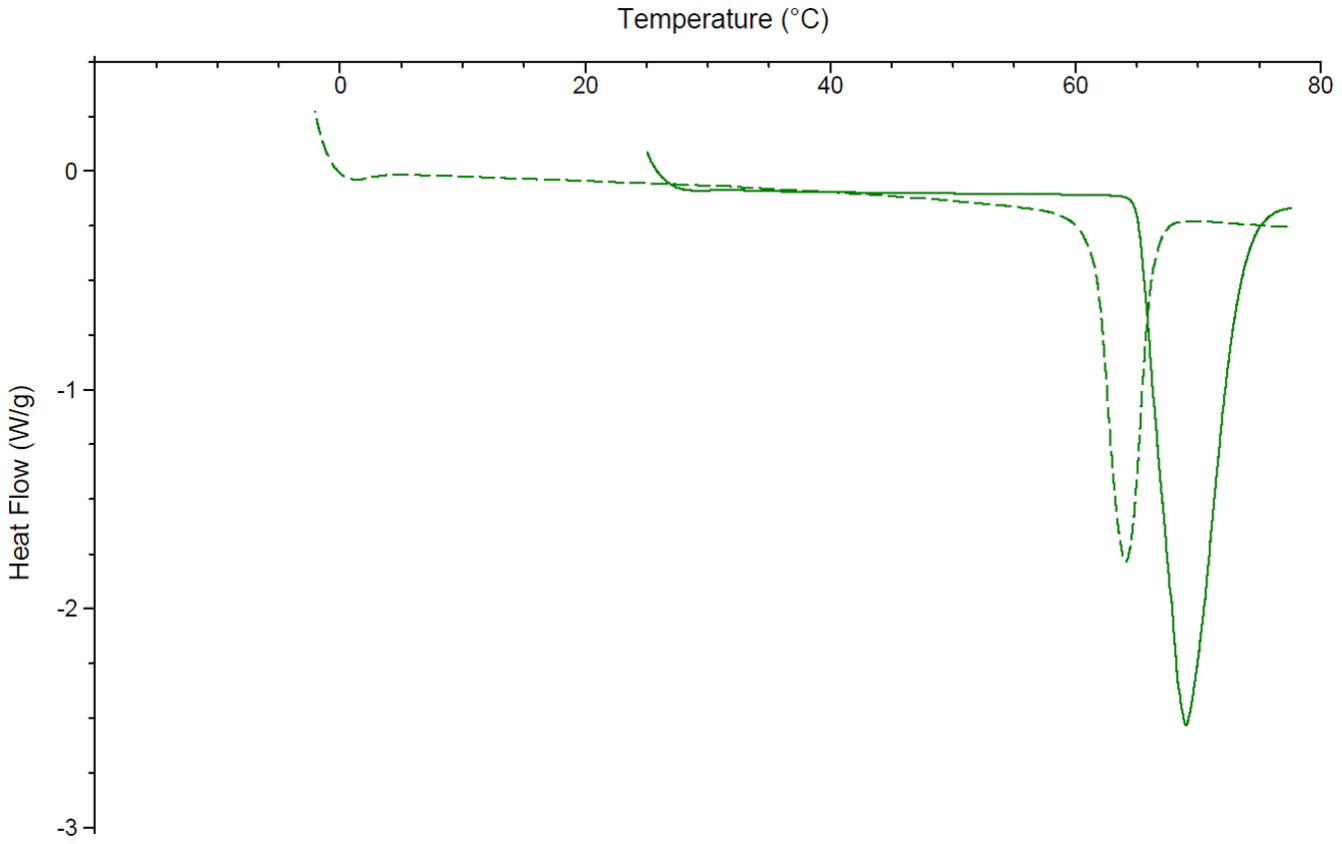
The differential scanning calorimetry curves of chlorocresol (solid line) and chlorocresol ground with CB[6] in a 50:50 w/w ratio (broken line).

**Figure 3:**
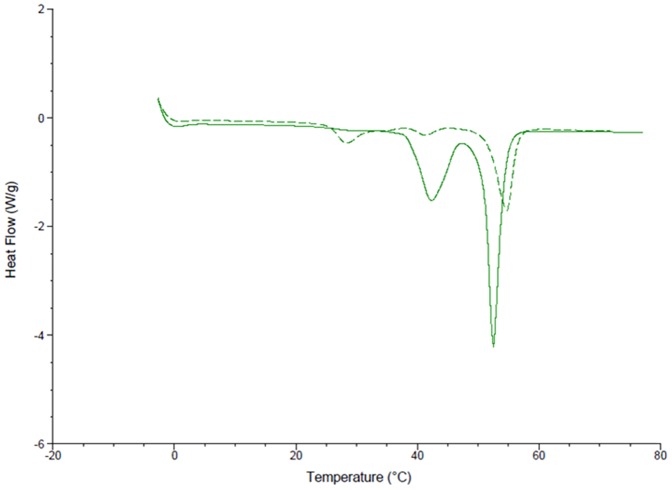
The differential scanning calorimetry curves of cetostearyl alcohol (solid line) and cetostearyl alcohol ground with CB[6] in a 50:50 w/w ratio (broken line).

Significant solid state interactions were observed between CB[6] and most excipients. Consistent with the literature, the DSC of chlorocresol shows a peak at 69°C with an enthalpy of fusion of 146.7 J/g (Fig. 2) [38]. The addition of CB[6] resulted in a reduction in the melting point to 64°C and a decrease in the peak size to 62.76 J/g. Cetostearyl alcohol has a melting range from 48 – 56°C [38]. In this study two peaks are observed at 43 and 53°C, the latter with an enthalpy of fusion of 115.1 J/g (Fig. 3). The DSC of cetostearyl alcohol with the addition of CB[6] displayed significant changes including a new peak at 27°C, a drop to a lower temperature of the peak previously at 43°C, a slight increase in the melting point of the major peak to 55°C and a significant drop in its enthalpy by 54% to 62.03 J/g.

**Figure 4:**
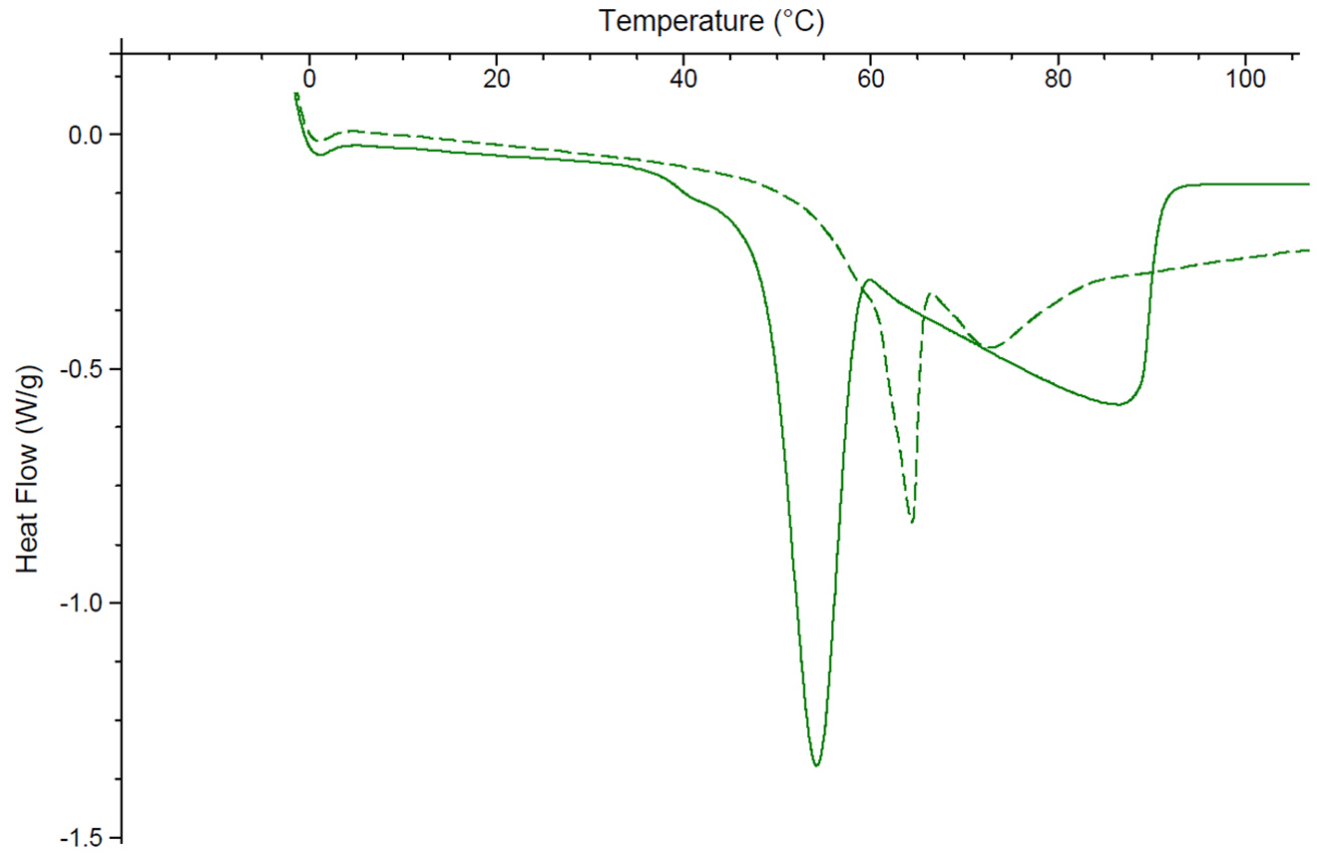
The differential scanning calorimetry curves of citric acid monohydrate (solid line) and citric acid monohydrate ground with CB[6] in a 50:50 w/w ratio (broken line).

Two DSC peaks were observed with citric acid monohydrate; one distinct peak at 54°C and another broad peak between 30 and 95°C (Fig. 4). While the addition of CB[6] causes the first peak to shift up to a higher temperature (64°C), the second peak remains relatively unchanged. To identify which peak accounted for the release of a water molecule and which the melting of the citric acid, samples of citric acid monohydrate and citric acid monohydrate ground in a 50:50 w/w ratio with CB[6] were analysed by TGA. The TGA results for citric acid monohydrate demonstrate a 5% weight loss at 30–50°C. For the citric acid-CB[6] mixture a 7% weight loss between 30–60°C is observed. From these results we can assume that the first peak (54/64°C) most likely coincides with the loss of water from both the citric acid and the waters of hydration of the cucurbituril and explains its movement to a higher temperature. The second broad peak was therefore assigned as the melting of the citric acid which has previously been shown to occur around 75°C [38]. Its drop from 88°C to 72°C indicates a significant interaction with the CB[6].

**Figure 5:**
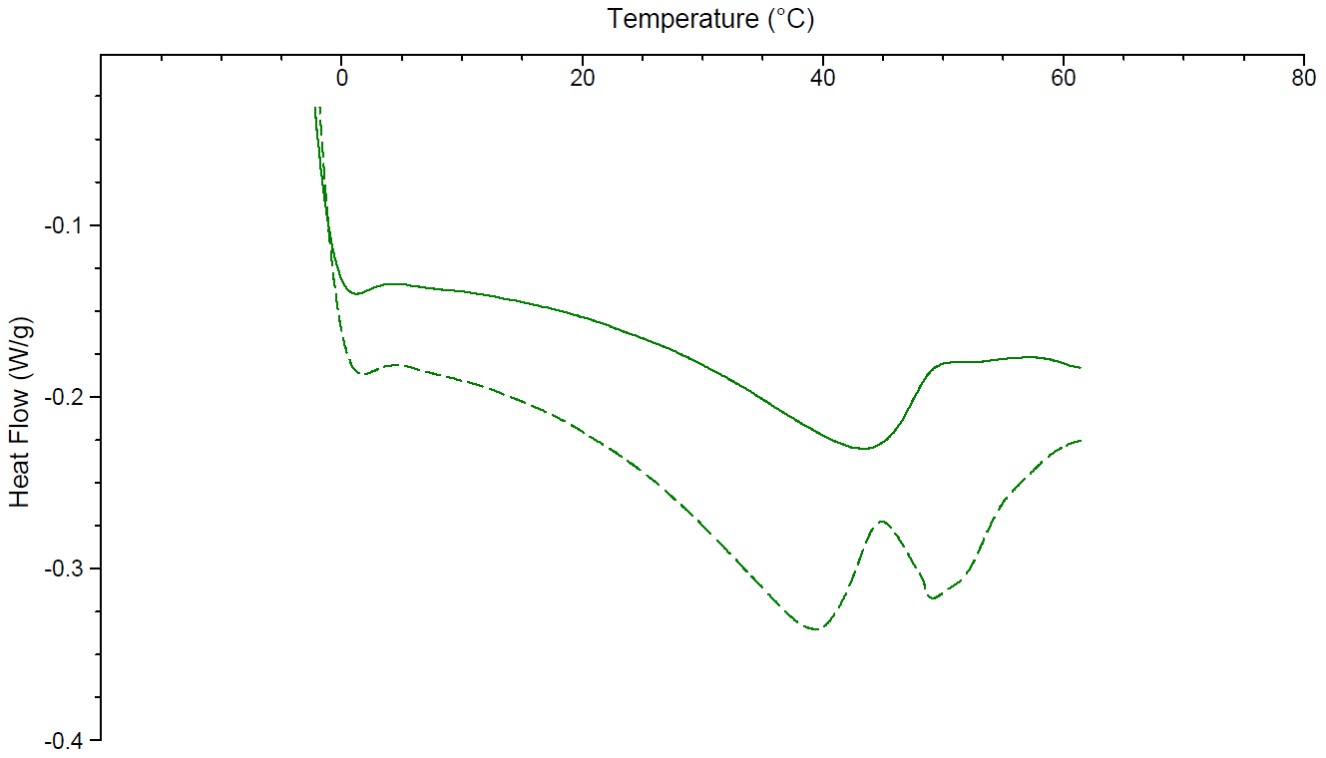
The differential scanning calorimetry curves of white soft paraffin (broken line) and white soft paraffin ground with CB[6] in a 50:50 w/w ratio (solid line).

**Figure 6:**
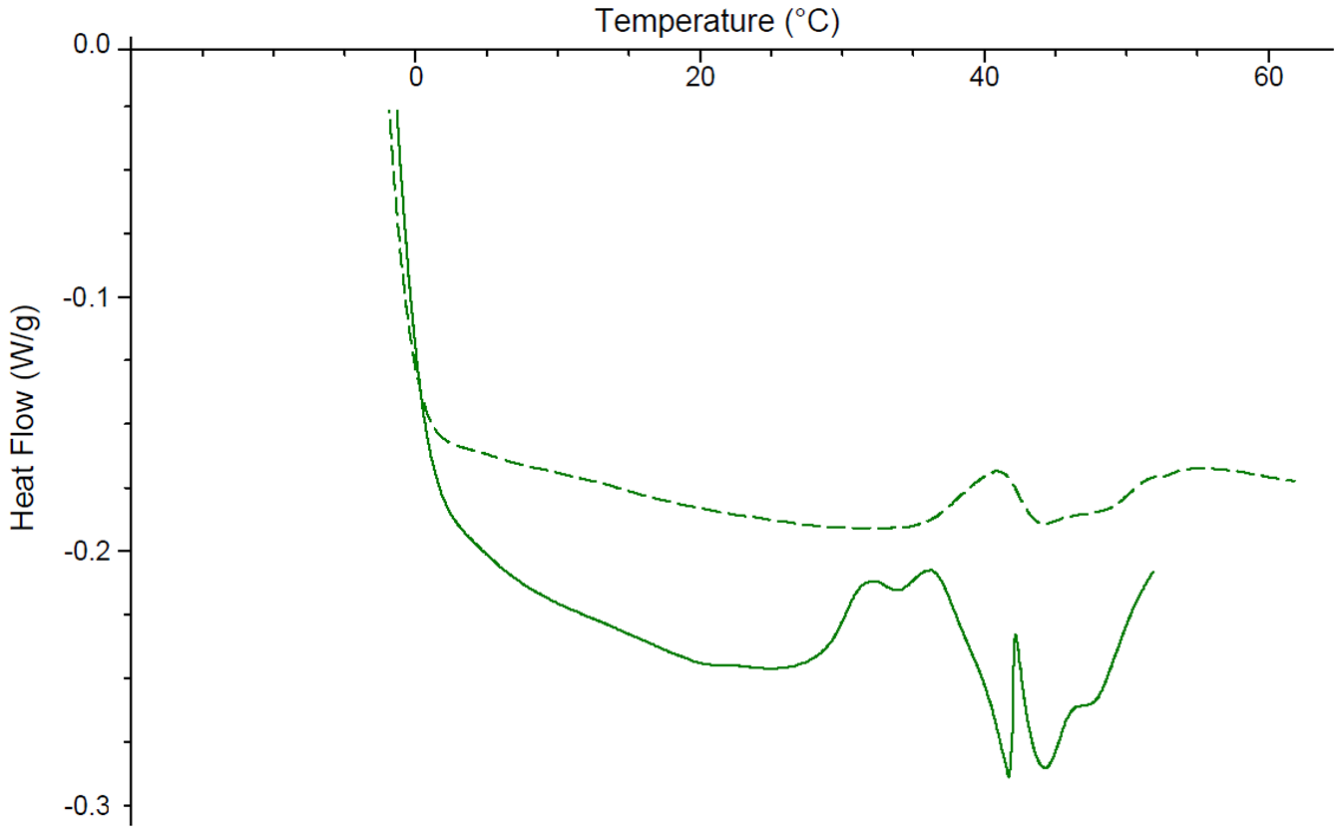
The differential scanning calorimetry curves of lanolin anhydrous (solid line) and lanolin anhydrous ground with CB[6] in a 50:50 w/w ratio (broken line).

The DSC of white soft paraffin shows two peaks between 10 and 45°C, and 45 and 60°C (Fig. 5). When ground with CB[6] the two peaks combine into one distinct peak at 43°C. The enthalpy of fusion could not be accurately determined as it was difficult to distinguish the start and end point of the peaks. The DSC of lanolin anhydrous displays three peaks between 38 and 52°C (Fig. 6). The addition of CB[6] results in a positive peak at 42°C which may suggest a glass transition occurs at this point. Grinding CB[6] with lanolin anhydrous also shifts the three peaks slightly up to 41–55°C with much less distinction between the peaks.

Overall, the results demonstrate that the CB[6] has measurable solid-state interactions with many of the excipients in the BCA and oily cream formulations; however, it is impossible to determine whether these interactions would impact the performance of the CB[6] and subsequent drug host-guest complexes.

### Formulation of CB[6] into topical cream-based dosage forms

Prior to examining CB[6] skin permeability, it was first necessary to assess the ability of CB[6] to be formulated into different topical dosage forms. Additionally, the maximum amount of CB[6] that could be formulated into each dosage form and still produce an effective product was investigated. Two topical creams were chosen as base formulations to investigate these aims. Aqueous-based and oily-based creams were selected to assess which formulation would better incorporate and deliver CB[6] into, and possibly through, skin. These creams included buffered cream aqueous (BCA) and an adaptation of the zinc cream oily formulation, both described in the APF22.

### Preparation of cream formulations

Cucurbit[6]uril was formulated into the creams by two methods: either by dissolving in the aqueous phase of each cream or incorporation into the prepared cream as a powder. Initially, attempts to dissolve CB[6] in the aqueous phase of the BCA were unsuccessful as the presence of chlorocresol with CB[6] resulted in the formation of an insoluble precipitate. Because of this chlorocresol is an incompatible excipient with CB[6] in liquid and semi-solid dosage forms. Instead, phenoxyethanol was selected as an alternate, which is a common preservative utilised in many topical dosage forms [39]. Subsequent dissolution experiments of CB[6] in the aqueous phase of BCA using phenoxyethanol did not result in any observable recrystallisation of CB[6] out of the solution by eye or under an optical microscope.

**Figure 7:**
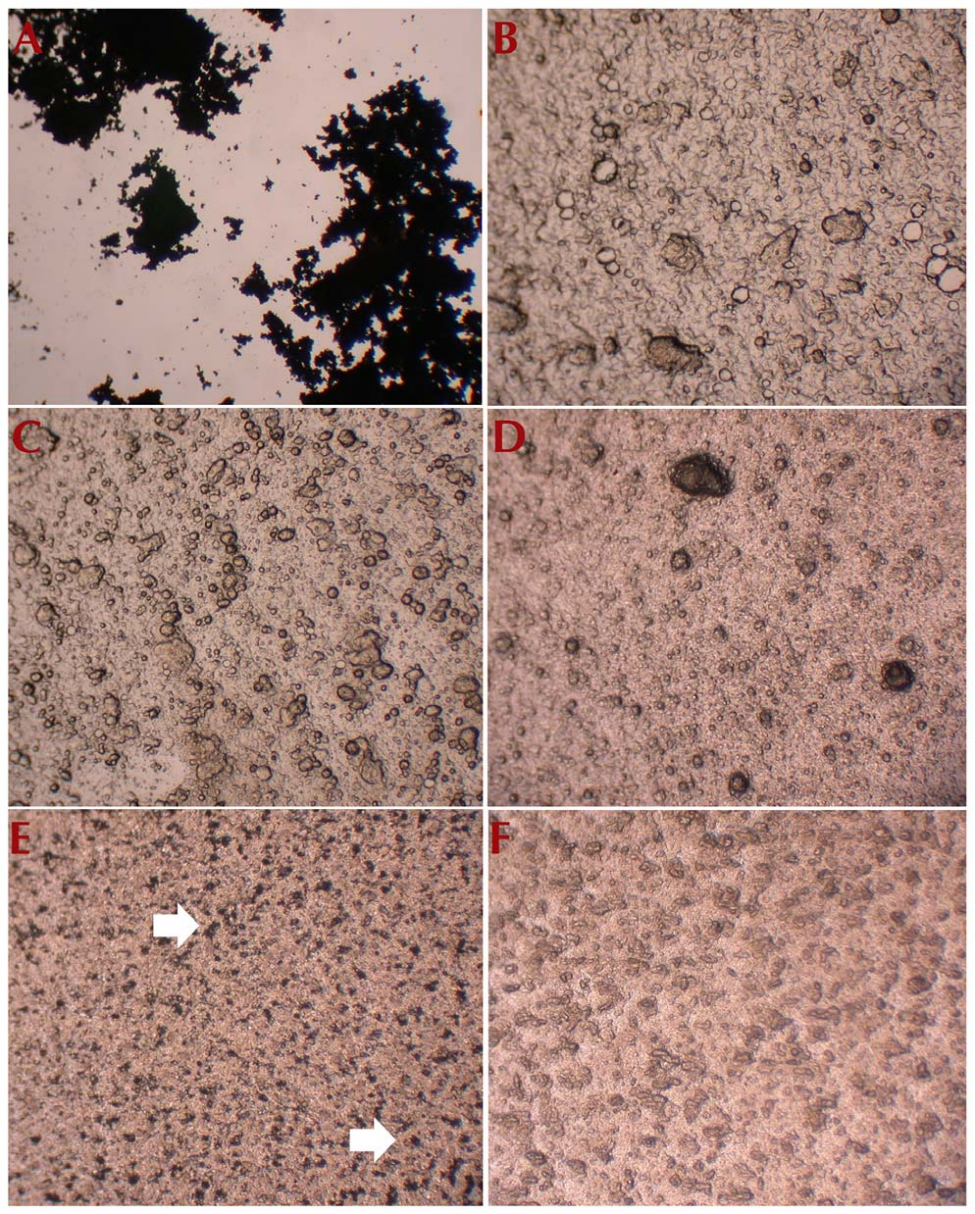
Optical microscope images of (A) CB[6] ground to a fine powder, (B) buffered cream aqueous with phenoxyethanol as the preservative, with no added CB[6], (C) 5% w/w CB[**6**] dissolved in the aqueous phase of BCA with phenoxyethanol, (D) 2–4% w/w CB[6] dissolved in the aqueous phase of BCA and mixed with 5 g of emulsifying ointment only, (E) 32% w/w CB[6] powder incorporated in buffered cream aqueous showing solid CB[6] particles in the formulation, and (F) 32% w/w CB[6] powder incorporated in oily cream. The latter panel (F) shows that the CB[6] dissolves in the oily cream completely.

Once formulated in BCA it was important to determine if CB[6] retained its solubility. Figure 7 shows optical microscope images of CB[6] and formulations containing CB[6]. When the CB[6] concentration is 2–4% w/w dissolved in the aqueous phase of the BCA and mixed with 5 g of emulsifying ointment, the resulting formulation does not display any CB[6] crystals. This may suggest that CB[6] remains in solution even when the CB[6] content in the aqueous phase is relatively high compared to the lipophilic phase (since the lipophilic phase should be 30 g according to the APF22). The absence of observable CB[6] crystals in Figures 7C and 7D may however suggest that either an insufficient amount of CB[6] was added to the cream to be detected visually or that the crystals remain in solution.

Attempts to dissolve CB[6] in the aqueous phase of the oily cream, which consisted of calcium hydroxide solution (30.5 mL) on a hotplate at >80°C with stirring were unsuccessful even after several hours. As a result, CB[6] was only able to be incorporated into the oily cream as a powder.

Observing the samples under an optical microscope also allowed for the detection of differences between formulating CB[6] into the BCA via dissolution in the aqueous phase and incorporation as a powder. Figures 7E and 7F illustrate the incorporation of CB[6] powder in the BCA and the oily cream at a concentration of 32% w/w, respectively. Figure 7E shows the presence of observable CB[6] crystals in the BCA formulation which indicates that CB[6] remains in the solid state in the cream and does not dissolve in the final product. In contrast, Figure 7F does not display any observable CB[6] crystals when incorporated as a solid into the oily cream even at the high concentration of 32% w/w. This indicates that CB[6] dissolves in the oily cream; despite not being soluble in the aqueous phase of the cream before production.

### Maximum CB[6] content formulated into cream

The maximum amount of CB[6] that can be dissolved in the aqueous phase of BCA and incorporated as a powder into the creams was investigated. This was done by adding CB[6] in 50 mg increments into the aqueous phase of BCA until solution saturation. It was found that 0.5% w/w (5.3 mM) CB[6] is the maximum amount that can be dissolved in the aqueous phase of BCA. This result is consistent with the solubility of CB[6] in other highly ionic solutions such as simulated gastric fluid (1–4 mM) and simulated intestinal fluid (5–7 mM) [6]. By comparison, in pure water CB[6] has a solubility of less than 0.02 mM [1]. All concentrations higher than 0.5% w/w resulted in observable amounts of recrystallised CB[6] upon cooling of the solutions. At 0.5% w/w no recrystallisation of CB[6] was observed even after the cream was completely formulated as indicated by the absence of CB[6] crystals in Figure 7C.

The maximum concentration of CB[6] that could be incorporated as a powder was measured by the amount of powder that can be absorbed into the skin, such that no observable powder or white residue remains on the surface of the skin. The zinc cream oily however contains 32% w/w zinc oxide powder and it was necessary to keep this fraction constant as altering the proportion of powder in the formulation may modify the properties of the cream. Whilst concentrations of CB[6] in the BCA of between 0.5 and 32% w/w has no obvious effects on the viscosity of the cream, the addition of CB[6] in oily cream at a concentration of 32% w/w resulted in a highly viscous and stiff product which was difficult to apply. As such, the oily cream was evaluated as an unsuitable formulation for the delivery of CB[6] and potentially other sized cucurbiturils.

### Skin permeability

Although human skin samples are ideal for permeability investigations they are not easily accessible. Nonetheless, studies have reported porcine [40]–[42] and rat skin [43]–[45] as good models for human skin since they exhibit similar anatomical and morphological characteristics. In this study skin from Australian albino white (AAW) rats was used as a suitable model.

Before skin permeation studies were conducted the ability of CB[6] to deposit into the skin was assessed by determining if any white residue remained on the skin surface after sample application. Each sample was applied using a spatula over a 4 cm^2^ area using BCA (5 mg) and BCA with 32% w/w CB[6] (5 mg). Upon examination of all samples, no observable powder or white residue remained on the surface of the skin, even after several hours. These results may suggest that CB[6] is able to penetrate into skin, although it cannot be discounted that the CB[6] does not penetrate the skin and human eyesight is too poor to detect CB[6] crystals on the skin surface visually.

**Figure 8:**
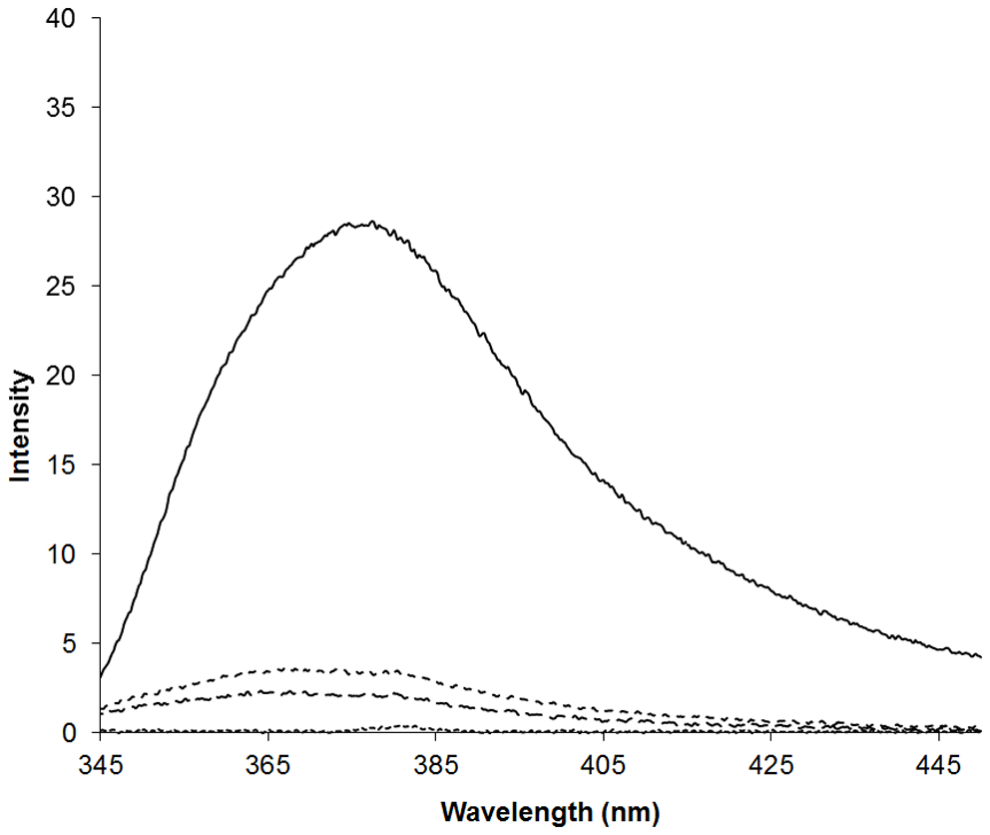
The fluorescence spectra of the free fluorescence marker (solid line), the fluorescent marker encapsulated by CB[6] (two dashed lines) and in water (dotted line).

The transport of CB[6] through skin was then examined with fluorescently marked CB[6] using Franz cells with a PBS reservoir. The free fluorescent marker 2,2′-(hexane-1,6diyl)diisoquinolinium in a 20% w/w BCA formulation is able to readily permeate through the skin. After 1 h the fluorescent intensity of the PBS solution is 28 which gave a fluorescent marker concentration of 2 µM in the reservoir (Fig. 8). When the fluorescent marker is encapsulated within CB[6] the hydrophobic environment increases the fluorescence of the marker by 25% and shifts the maximum to a lower wavelength [7]. When encapsulated by CB[6] the amount of fluorescent marker that is able to permeate through the skin is significantly lower. Cucurbit[6]uril encapsulation results in a fluorescence intensity drop to between 2–3 which is a decrease of 92%. When fluorescence enhancement by CB[6] of the marker is taken into account that the actual amount of CB[6]-fluorescent marker that has permeated through the skin is just 6% compared with the free fluorescent marker. This result suggests that CB[6] does not permeate easily through skin in the BCA formulation and therefore may have more application in localised skin treatment than it does for transdermal drug delivery applications. Potential applications could include the treatment of wounds or skin-based diseases such as psoriasis and eczema and it could be used to increase the residence times of the active pharmaceutical ingredients of sunscreens. It is important to note, however, that CB[6] is just one homologue from the cucurbituril family and the other sized cucurbiturils may display different skin permeability. Likewise, different topical dosage forms may also display different skin permeability for CB[6] and other homologues.

## Conclusions

The macrocycle CB[6] was formulated into buffered cream aqueous and an oily cream. Cucurbit[6]uril was shown to have solid state interactions with the excipients in both dosage formulations and when added to the oily cream produced a product too viscous and stiff to perform as an appropriate medicinal product. Cucurbit[6]uril in the BCA formulation does not permeate easily through skin, which means its drug delivery applications may be limited to localised treatments only. Further research using different sized cucurbiturils and with different topical dosage forms is warranted to determine if cucurbiturils can be used in transdermal applications.
